# Bullous cellulitis with bacteraemia caused by multi-drug resistant *Myroides odoratimimus* species

**DOI:** 10.1016/j.idcr.2026.e02797

**Published:** 2026-10-14

**Authors:** Rachel Perthiani, Peter Conlon, Kate Hanley, Clarice Egan, Eibhlin Higgins

**Affiliations:** a Department of Infectious Diseases, University Hospital Galway, Galway, Ireland; b Department of Microbiology, University Hospital Galway, Galway, Ireland; c College of Medicine Nursing and Health Sciences, University of Galway, Galway, Ireland

**Keywords:** Bullous cellulitis, *Myroides*, Multi-drug resistance

## Abstract

*Myroides odoratimimus* is an aerobic, non-fermenting notoriously multi-drug resistant bacillus which is ubiquitous in the environment, rarely implicated in human disease. We report a case of a man in his 60 s with multiple comorbidities who presented with a two day history of acute swelling, erythema and tenderness of the left leg and foot, associated with oozing, ulceration and bullous lesions. The blisters were serous filled and ruptured upon contact (see Figure 1). Blood cultures and wound cultures yielded the multi-drug resistant organism *Myroides odoratimimus*. Treatment is challenging due to extensive intrinsic antimicrobial resistance. Susceptibility testing confirmed sensitivity to meropenem and co-trimoxazole (see Table 1), the patient was treated with combination therapy resulting in rapid reduction of inflammatory markers and resolution of lesions (see Figure 2). This case outlines how cirrhosis / poorly controlled diabetes impacts the infection risk of the host and how infection with *Myroides* species may occur without a clear environmental exposure as well as the implications this organism may have on infection control measures.

## Introduction

The *Myroides* genus comprises non-motile gram negative bacilli widely distributed in soil and water environments. Formerly grouped with *Flavobacterium*, genotypic and phenotypic differences lead to their reclassification in 1996 [1]*.* In recent years infections with *Myroides* species have been increasingly reported in immunocompromised patients, in particular those with multiple comorbidities most notably chronic kidney disease (CKD), cirrhosis, diabetes, chronic obstructive pulmonary disease [2]. Infections that have been attributed to *Myroides* species include skin and soft tissue infections, urinary tract infections, osteomyelitis, and central nervous system infections. Two cases of intracranial *Myroides odoratimimus* infection have been described, one following external ventricular drains in an immunocompetent adult [3], [4]. *Myroides* is usually associated with a form of external inoculation such as the case outlined by Maraki et al. with a *Myroides* soft tissue wound infection following a pig bite in an immunocompromised child [5]. *Myroides* have a propensity to form biofilms as illustrated by a reported case of a *Myroides odoratimimus* infection of a recurrent calcaneal ulcer in an immunocompromised host [6]. The purpose of this case is to highlight the clinical significance of *Myroides* infection in patients with multiple comorbidities and the potential challenges in management of patients with this highly antibiotic resistant organism. This case provides practical insights into the importance of timely diagnosis, utility of advanced microbiologic diagnostics and directed therapy to improve patient outcomes. It outlines a rare presentation of *Myroides odoratimimus* bacteraemia in a patient with bullous cellulitis and multiple comorbidities with no exposure to soil or contaminated water.

## Case presentation

This case describes a patient in his 60 s with a complex background comprised of type two diabetes mellitus with suboptimal glycaemic control (HbA1C on admission of 86 mmol/mol), CKD, Parkinson’s disease, coronary artery disease (post coronary artery bypass graft), non-severe proliferative diabetic retinopathy, cirrhosis and chronic pancreatitis. He has a family history of diabetes mellitus and ischaemic heart disease. The patient is a non-smoker with a previous history of alcohol misuse. He presented to the Emergency Department with a two day history of acute swelling, erythema and tenderness of the left leg and foot, associated with oozing ulceration and bullous lesions (see Fig. 1). The oedematous, erythematous and painful bullous lesions limited his mobility. He denied any environmental exposure or any foreign travel recently. The patient had no recent hospital admissions, invasive procedures, indwelling catheters prior to the admission. He denied dysuria, urinary frequency and flank pain. Urinalysis was performed and was unremarkable, making a urinary source unlikely. A physical examination did not identify any alternative infective focus other than the visible healing diabetic left foot ulcer and left lower limb cellulitis. Although multiple healthcare contacts were involved given the chronic comorbidities, the evidence was insufficient to definitively classify the stated infection as healthcare-associated. The clinical impression at the time of admission was of bullous cellulitis. The patient had a wound swab taken and was initially started on IV flucloxacillin 2 g four times a day. (Table 1)

**Fig. 1:**
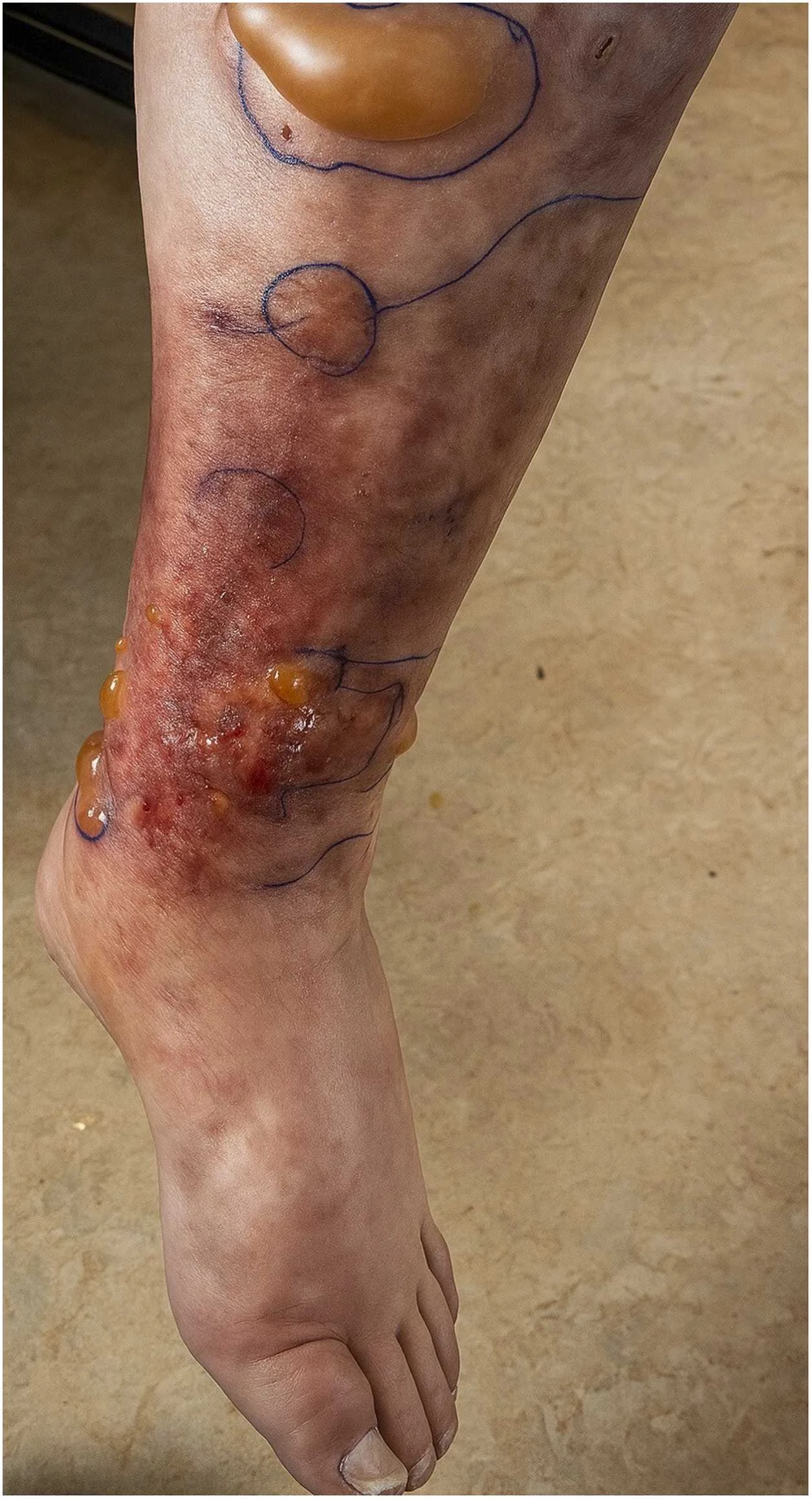
Extensive bullous cellulitis of the left lower limb, oozing tense serous-filled bullae with surrounding oedema and erythema prior to lancing.

**Fig. 2:**
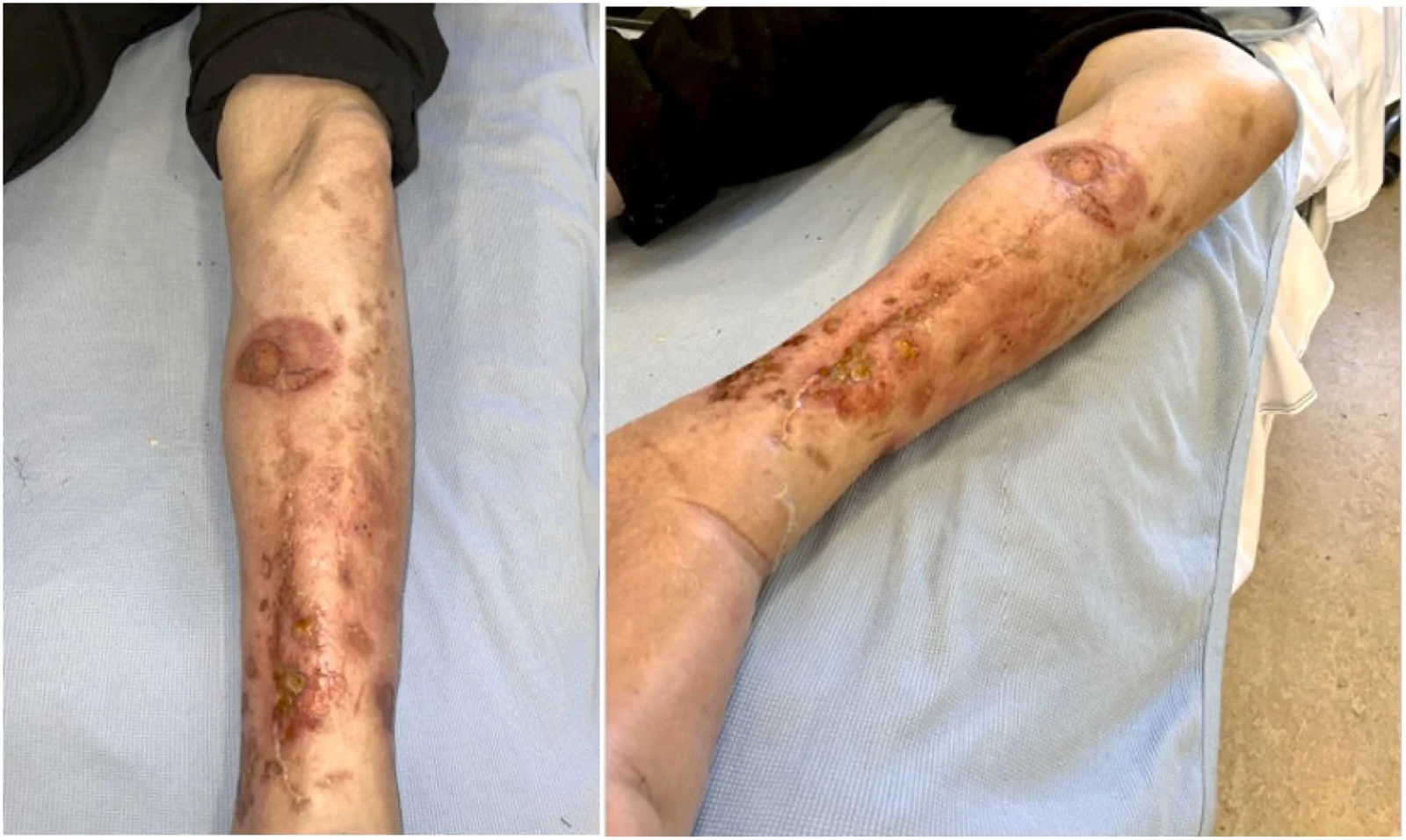
Appearance of lesions post lancing, demonstrating crusting and a decrease in erythema.

**Table 1:** Antimicrobial susceptibility profile of Myroides odoratimimus isolated from blood cultures, demonstrating multidrug resistance with retained susceptibility to co-trimoxazole and meropenem.

Antibiotic	MIC (μg/mL)	Interpretation
*Penicillin*	*6.0*	*R*
*Piperacillin-Tazobactam*	*48.0*	*R*
*Tigecycline*	*1.0*	*R*
*Co-trimoxazole*	*1.0*	*S*
*Ciprofloxacin*	*> 32.0*	*R*
*Meropenem*	*1.5*	*S*
*Ceftriaxone*	*> 32.0*	*R*
*Ceftazidime*	*64.0*	*R*
*Tetracycline*	*> 256.0*	*R*

Initial laboratory results included C reactive protein 332 mg/L (0−5), white cell count 22.6 × 10^9^ (range 4–10), neutrophils 18.61 × 10^9^ (range 2–7), platelets 96 × 10^9^ (range 150–400), creatinine 229 umol/L (range 64–104) (eGFR 24 mL/min/1.73m^2^).Blood cultures yielded gram negative bacilli subsequently identified as *Myroides odoratimimus.* Initial susceptibilities demonstrated resistance to gentamicin and ciprofloxacin. Wound swab cultures also isolated *Myroides odoratimimus* hence identifying the causative organism in the setting of both wound and blood cultures supported this organism as the causative pathogen*.* Antimicrobial therapy was subsequently escalated to IV piperacillin-tazobactam followed by combination therapy with IV meropenem and PO co-trimoxazole. Combination therapy was utilized pending final results of antimicrobial susceptibility testing. Topical treatment of the skin included paraffin gel as an emollient and topical corticosteroid with betnovate ointment.

During the course of his admission he reported worsening low back pain and an MRI of the lumbar and sacral was undertaken to evaluate for spondylodiskitis due to a concern for potential haematogenous seeding. This demonstrated degenerative changes with multilevel foraminal stenosis but no evidence of infection. By hospital day 10 his CRP had decreased from 332 to 5.7 with significant associated clinical improvement. He completed a 14 day course of antimicrobial therapy and was subsequently discharged.

## Investigations

### Microbiology

#### Microscopic, macroscopic appearance and biochemical testing

*Myroides* species grow on a variety of media, including MacConkey agar (see Fig. 3).Growth typically occurs between 18°C and 37°C, but not at 41°C. Colonies are characteristically yellow and emit a fruity odour [2].These organisms are oxidase negative, urease negative and gelatinase positive. They reduce nitrite reductase and do not produce indole [7], [8]

**Fig. 3:**
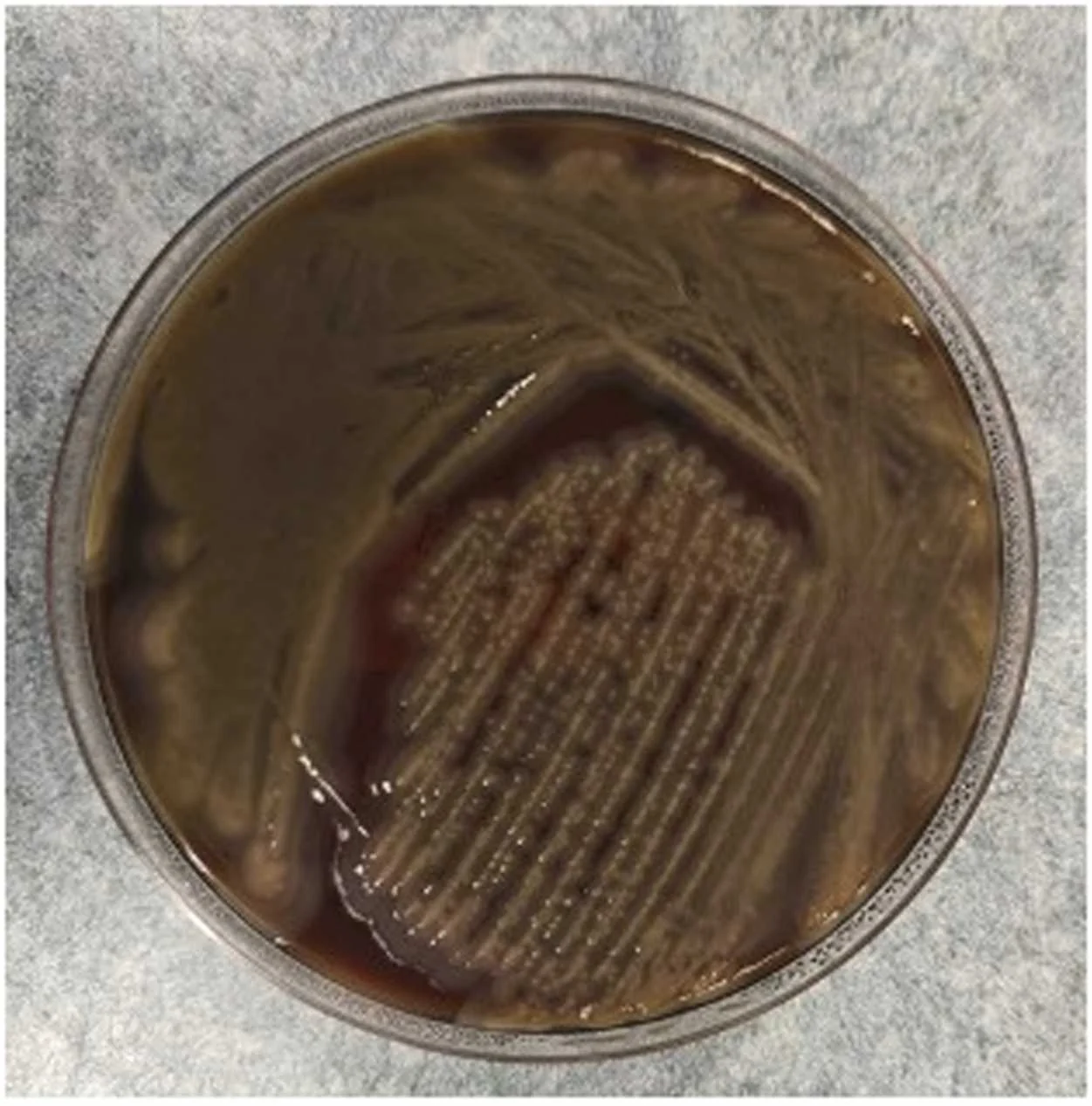
Growth of Myroides odoratimimus on chocolate agar with characteristic yellow-pigmented colonies.

#### Species identification

The isolate was identified as *Myroides odoratimimus* using the bruker matrix-Assisted laser desorption/ionisation time-of-flight (MALDI-TOF) mass spectrometry biotyper sirius system, comparing protein spectra of isolates to a reference database for precise species level identification.

#### Antibiotic susceptibility testing

Performed using Epsilometer tests (E-tests).This method uses a plastic strip impregnated with a predefined gradient of an antimicrobial, enabling a quantitative determination of the minimum inhibitory concentration (MIC). The strip is placed on an agar plate already seeded with the organism of interest. As the antibiotic diffuses into the agar, an elliptical zone of inhibition forms, and the MIC is read at the point where growth intersects the strip’s scale [9]. MIC results were interpreted according to the european committee on antimicrobial susceptibility testing (EUCAST) guidance on antimicrobial agents for treating aerobic bacteria without EUCAST breakpoints [10]

Wound swab: *Myroides odoratimimus*

Blood cultures (aerobic and anaerobic): *Myroides odoratimimus*, resistant to gentamicin and ciprofloxacin

## Differential diagnosis

Bullous cellulitis *secondary to Myroides odoratimimus* was considered due to the presence of tense bullae on the lower limbs, erythema, tenderness and warmth which are the classic features of bullous cellulitis (see Fig. 1).This diagnosis was supported further with the positive wound and blood cultures confirming *Myroides odoratimimus*. This patient’s immunocompromised status had also increased the susceptibility for this opportunistic pathogen.

Polymicrobial diabetic foot infection was considered based on the patient’s background of poorly controlled diabetes. However the ulcer over his left first metatarsophalangeal joint had improved compared to recent outpatient review, an MRI foot did not demonstrate any evidence of underlying osteomyelitis and the nature of his presentation was atypical for a spreading cellulitis from a diabetic foot infection.

Autoimmune blistering disorder such as pemphigus vulgaris or bullous pemphigoid can present with flaccid or tense bullae. Serological testing for antinuclear antibody, antineutrophil cytoplasmic antibody, and anti-cyclic-citrullinated peptide were negative. With positive microbiologic results and clinical features favouring infection it was felt that an autoimmune cause was unlikely and a skin biopsy was deferred.

The patient had an underlying vascular insufficiency and while peripheral arterial disease can cause lower extremity ulcers with delayed healing, the timeframe, clinical characteristics and distribution of skin changes were not consistent with ischaemic changes. The final diagnosis was made based on the combination of clinical characteristics consistent with bullous cellulitis, supportive microbiologic data and exclusion of alternative causes.

## Treatment

Initial therapy: Flucloxacillin 2 g IV four times daily

**Definitive treatment**: Total 14 day antimicrobial therapy course initially with co-trimoxazole 960 mg twice daily and meropenem 1 g IV three times daily and subsequently IV meropenem 1 g IV three times daily.

Non-pharmacological management: Lancing of large bullae**,** Topical therapy (paraffin gel, betnovate)**,** Podiatry: management of diabetic foot ulcer**;** Supportive care: IV fluids, wound care **,** pregabalin for diabetic neuropathy (100 mg morning, 125 mg nocte)

## Outcome and follow-up

Evaluation and management of this complex case required multidisciplinary input from microbiology, infectious diseases, endocrinology, podiatry and dermatology. With collaborative care and appropriate antimicrobial therapy he achieved significant clinical improvement and was ultimately fit for discharge by hospital day 14.

## Discussion

Two most common *Myroides* species seen in humans are *Myroides odoratus* and *Myroides odoratimimus* [11], Most prior reports are linked to environmental exposure, or indwelling devices. In contrast our patient had no such exposure, suggesting that cirrhosis and diabetes alone can be significant predisposing factors. A retrospective cohort study showed the most common infections were skin/soft tissue (42.8%), osteomyelitis (33.3%), and urinary tract infections; 28.5% were bacteraemia [12], All isolates were resistant to aminoglycosides but susceptible to meropenem, ciprofloxacin,trimethoprim-sulfamethoxazole [12].This case demonstrates the pathogenic potential of *Myroides* in skin and soft tissue and highlights that there may not be an apparent environmental exposure.

This case represents a presentation of *Myroides odoratimimus* with associated bullous cellulitis and concurrent bacteraemia in a patient with cirrhosis and poorly controlled diabetes*.* The source was confirmed with both wound swab and blood cultures positive for *Myroides odoratimimus,* demonstrating concordance. As for susceptibility despite the resistance to aminoglycosides and fluoroquinolones the isolate retained susceptibility to meropenem and co-trimoxazole. This combination therapy was selected based on the severity of infection, expected susceptibility profile and the patient’s significant comorbidity burden. This case highlights the clinical value of extended antimicrobial sensitivity testing in pathogens like *Myroides.odoratimimus.*

Taking into account the *Myroides* species intrinsic multidrug resistance profile and their ability to survive in hospital settings as well as environmental reservoirs, isolation of this organism has important infection prevention and control considerations. Recent literature has identified Myroides species as an emerging nosocomial pathogen. Aygar et al. described a series of 67 patients with Myroides odoratimimus isolates across a five-year period, highlighting clonal transmission within hospital settings, particularly in intensive care unit patients with multiple comorbidities [13]. Karvar et al. described a 27-patient clonal ICU outbreak and noted five hospital-based outbreaks in the literature. Common features included occurrence in intensive care settings, the absence of an environmental source, as well as extensive antimicrobial resistance. Molecular typing showed clonal relatedness between isolates increasing the risk of patient-to-patient transmission [14]. There was no temporal relationship in this case with an inpatient admission to suggest healthcare associated acquisition and there were no other cases identified.

Most reported cases of Myroides described had required a form of direct inoculation such as that by Meyer et al. where a lower extremity cellulitis and bacteraemia had occurred following contact with soil and exposure to dog saliva in a diabetic host, distinctly our patient had no environmental or animal exposure [15]. A majority of cases manifest as non-bullous cellulitis or chronic non-healing ulcers. For example, Encicott et al. had described of a Myroides odoratimimus leg ulceration and cellulitis without systemic spread and Beathard et al. reported of a reporting of non bullous Myroides cellulitis without vesicle or bulla formation in an immunocompromised patient with chronic renal failure and type two diabetes [16], [17]. In sharp contrast our patient developed tense serous bullae with frank rupture and concurrent Myroides bacteraemia further favouring an atypical dermatological manifestation. Both cases illustrate together how host comorbidities such as liver cirrhosis, chronic renal failure and poorly controlled diabetes can facilitate an opportunistic Myroides infection in the absence of an environmental exposure. Further taking into account key shared host risk factors Lovering et al. described a case of cellulitis secondary to a small puncture wound from a crab shell occurring in an elderly male with compensated cirrhosis, highlighting liver cirrhosis as a shared risk factor and distinctly contrasts from a mechanism standpoint, further depicting how severe endogenous metabolic dysfunction in particular, combined liver cirrhosis and poorly controlled diabetes can impair cutaneous barrier immunity [18].

The pathogenicity of *Myroides odoratimimus* is attributed to its ability to form biofilms and produce metalloproteases, enhancing tissue invasion [6]. These bacteria thrive in high concentration glucose environments and suboptimal glycaemic control was likely a significant predisposing factor in this case in addition to underlying cirrhosis [2], [19]. *Myroides* soft tissue infections and cellulitis have been reported, however descriptions of tense serous bullae with frank rupture are not a well-emphasised feature in most prior reports. Given the significant antimicrobial resistance profile associated with this organism, standard recommended regimens for soft tissue infections would likely not provide adequate antimicrobial coverage, highlighting the importance of appropriate microbiologic testing before commencing antibiotics as well as considering atypical pathogens in patients not improving on standard antimicrobial therapy. This case puts an emphasis on clinicians to consider *Myroides* in immunocompromised patients who present with atypical bullous cellulitis*.*

What distinguishes this case from the already existing Myroides literature is the combination of clinical, epidemiologic, microbiologic and therapeutic factors, Clinically the patient presented with rapid tense serous bullae with associated oozing, this represents an atypical blistering phenotype which in existing reports manifests as chronic ulcers or non-bullous cellulitis [16], [17]. Epidemiologically the high-grade bacteraemia and invasive nature occurred in the absence of any recognized soil, animal or healthcare-associated exposures, depicting how combined poorly controlled diabetes and liver cirrhosis can drive systemic invasion without an external inoculation. Microbiologically M.odoratimimus was isolated in peripheral blood cultures and deep wound swabs, concordance supports this as the causative pathogen in this case. Myroides species are known for extensive antimicrobial resistance this strain was resistant to tetracyclines but susceptible to meropenem and co-trimoxazole. Therapeutically there was a distinction as severe Myroides soft tissue infections can require surgical debridement, in this case the patient achieved a rapid inflammatory marker improvement using targeted antimicrobials as well as bedside lancing (see Fig. 2).

## Learning points

*Myroides* species can cause invasive infection even without environmental exposure

Extended susceptibility testing is crucial as resistance patterns are highly variable.

With targeted therapy good outcomes are achievable in even frail, comorbid patients as evident in this patient. This case adds to the small amount of literature on *Myroides odoratimimus* soft tissue infections with bacteraemia highlighting in particular the bullous cellulitis presentation, complex susceptibility profile and successful management with multidisciplinary care.

## Patient’s perspective

***“**I don’t want another person to go through what I went through so I’d like to explain what I felt before I was admitted into the hospital. For two days my leg was very painful and warm. It takes me exactly thirty three steps to get to my room in the hostel and I realised it became difficult for me to take those steps. I hoped it would go away on its own so I delayed treatment for those forty-eight hours. I never experienced such an insane pain in my life. I initially thought it was a rash and didn’t think much of it and turned the other way. My advice would be to go straight to the doctor and straight to hospital no matter what age you are, otherwise you’ll end up like me. I couldn’t cope with the agony but I stayed positive and I stayed hopeful throughout.**”***

## Intellectual property rights assignment or licence statement

I, **[RACHEL PERTHIANI]**, the Author has the right to grant and does grant on behalf of all authors, an exclusive licence and/or a non-exclusive licence for contributions from authors who are: I) UK Crown employees; ii) where BMJ has agreed a CC-BY licence shall apply, and/or iii) in accordance with the relevant stated licence terms for US Federal Government Employees acting in the course of the their employment, on a worldwide basis to the BMJ Publishing Group Ltd (“BMJ”) and its licensees, to permit this Work (as defined in the below licence), if accepted, to be published in BMJ Case Reports and any other BMJ products and to exploit all rights, as set out in our licence author licence.

I obtained formal consent from the competent patient who agrees for their data to be published. The patient consented to images and data to be uploaded for medical research purposes.

## CRediT authorship contribution statement

**Rachel Perthiani:** Writing – review & editing, Writing – original draft, Investigation, Conceptualization, Data curation, Formal analysis, Methodology, Visualization, Resources. **Peter Conlon:** Conceptualization. **Kate Hanley:** Visualization. **Clarice Egan:** Investigation. **Eibhlin Higgins:** Writing – review & editing, Supervision, Methodology.

## Ethics declaration

Written informed consent to take part in the study and to publish the article has been obtained from all participants or their legal representatives. The privacy rights of participants have been observed.

This study was performed in compliance with relevant laws, regulatory frameworks and guidelines where the research took place. Ethics committee approval was not required under relevant laws and institutional guidelines. Informed consent was obtained from the competent patient

This research follows the CARE guidelines and the CARE checklist.

## Declaration of Competing Interest

The authors declare that they have no known competing financial interests or personal relationships that could have appeared to influence the work reported in this paper
